# Optimized miR-124 reporters uncover differences in miR-124 expression among neuronal populations *in vitro*

**DOI:** 10.3389/fnins.2023.1257599

**Published:** 2023-10-18

**Authors:** Catherine Lepolard, Cynthia Rombaut, Florence Jaouen, Ana Borges, Elodie Caccomo-Garcia, Natalia Popa, Eduardo Gascon

**Affiliations:** ^1^Aix Marseille University, CNRS, INT, Institute of Neuroscience of la Timone, Marseille, France; ^2^Neurobiotools Facility (Neurovir), Aix Marseille University, CNRS, INT, Institute of Neuroscience of la Timone, Marseille, France

**Keywords:** miRNA, miR-124 reporters, neuron, AAV, mice

## Abstract

**Introduction:**

Although intensively studied in the last decades, how microRNAs (miRNAs) are expressed across different cell types in the brain remains largely unknown.

**Materials:**

To address this issue, we sought to develop optimized fluorescence reporters that could be expressed in precise cellular subsets and used to accurately quantify miR contents *in vivo*.

**Results:**

Focusing on miR-124, we tested different reporter designs whose efficiency was confirmed in different *in vitro* settings including cell lines and primary neuronal cultures from different brain structures. Unlike previous reporters, we provide experimental evidence that our optimized designs can faithfully translate miR levels *in vitro*.

**Discussion:**

Tools developed here would enable assessing miRNA expression at the single cell resolution and are expected to significantly contribute to future miRNA research *in vivo*.

## Introduction

microRNAs (miRNAs) are a class of short (20–25 nt) non-coding RNAs (Huntzinger and Izaurralde, 2011; Izaurralde, 2015). miRNAs repress expression of messenger RNAs (mRNAs) containing short complementary sequences. Since these sequences could be found in hundreds of transcripts, miRNAs are thought to fine-tune gene expression at a global scale (Fabian and Sonenberg, 2012). Mechanistically, ithas long been known that miRNA-dependent silencing critically relies on the complementarity of miRNA-mRNA sequences. The complementarity in first eight nucleotides of the miRNAs (the so-called seed region) is critical for target recognition whereas mismatches in the remaining sequence are tolerated and do not seem to affect miRNA binding efficiency (Huntzinger and Izaurralde, 2011). Nonetheless, perfect base-pairing has been consistently shown to result in transcript degradation whilst translational inhibition is the main silencing mechanisms for those targets with lower degree of complementarity (Yekta et al., 2004; Valencia-Sanchez et al., 2006).

miRs have attracted much attention in the context of neurodegenerative diseases. On one hand, because a growing body of evidence suggest that miRNAs dysregulation is a key pathogenic event in multiple diseases (Liu et al., 2012; Gascon et al., 2014; Salta and De Strooper, 2017). On the other hand, because of their simultaneous action on different biological pathways, they hold a promising potential for therapeutic applications (Gentile et al., 2022; Seyedaghamiri et al., 2023).

miR-124 is one of the best studied miRNAs because of: (i) its abundance (Lagos-Quintana et al., 2002); (ii) conservation across species and brain enrichment (Lagos-Quintana et al., 2002); (iii) involvement in multiple key biological processes in the brain ranging from neuronal differentiation during development (Makeyev et al., 2007; Maiorano and Mallamaci, 2010) to fine-tuning neurotransmitter receptor composition at the synapse (Hou et al., 2015; Gilbert et al., 2016; Namkung et al., 2023); and (iv) alterations observed in a wide variety of pathological conditions, especially neurodegenerative diseases (Gascon et al., 2014; Wang et al., 2018; Yao et al., 2019; Ghafouri-Fard et al., 2022). Remarkably, there is currently no information about miR-124 expression at the single-cell resolution.

Owing to their small sizes and relative low abundance, miRNA quantification has been technically challenging. miRNA expression has been evaluated using *in situ* hybridization (Urbanek et al., 2015), northern blot (Kim et al., 2010), microarrays (Cissell and Deo, 2009), RT-PCR amplification (Chen et al., 2005) and, more recently, sequencing (small RNA sequencing) (Zhou et al., 2011; Podnar et al., 2014). However, except last-generation *in situ* hybridization with RNAscope plus probes, precise quantification and cellular resolution remains an unsolved problem. The advent of single-cell RNA sequencing (scRNA-seq) technology has enabled transcriptomic profiling of individual cells and enormously contributed to our understanding of brain complexity at the molecular level. Nevertheless, current scRNA-Seq methods are restricted to long RNAs, primarily mRNAs (Macosko et al., 2015; Rosenberg et al., 2018). In summary, due to technical constraints, how miRNAs are expressed in different cell types *in vivo* remains elusive.

To monitor miRNA levels, several groups have devised different reporter strategies [reviewed in (Song et al., 2020)]. Among them, miRNA binding sequence-engineered fluorescent reporters have been particularly attractive, as they might enable both precise quantification and visualization of miRNA level in living cells (Kato et al., 2010; Sano et al., 2017; Wang et al., 2019). In the same line, several transgenic reporter mice strains have been generated aiming at characterizing the cellular expression of different miRNAs in the brain *in vivo* (Åkerblom et al., 2012, 2013; Akerblom et al., 2014). Previous miRNA reporter constructs have been engineered to contain multiple (4 or more) perfectly complementary sequences for a particular miRNA. Such reporters are submitted to a strong inhibition *in vivo* so that only cells completely devoid of the miRNA show reporter expression (Åkerblom et al., 2012, 2013; Keaveney et al., 2018, 2020). These observations argue for a non-physiological regulation of this kind reporter. In summary, new reporters are therefore required to circumvent the above-mentioned technical hurdles.

We reasoned that mimicking endogenous targets should be more appropriate for research purposes. We sought to develop reporter designs based on more physiological binding sequences for miR-124. We choose miR-124 as a prototypical miRNA involved in multiple brain diseases. Setting up such miR-124 reporters could not only provide important insights into the generalization of our approach to other miRNAs but also, because of its conservation, it could serve to interrogate different species. Here, we investigated *in vitro* the activity of our novel reporters and compared them with previous designs. Using different cell lines and primary neurons, our results show that reporters bearing endogenous binding sequences are more reliable to evaluate miR-124 expression. Overall, we provided novel tools that can be useful for the investigation of expression profiles of specific miRNAs at the cellular resolution and, more importantly, that should be applicable to monitor/identify specific cell populations *in vivo*.

## Methods

### miR-124 reporter design

For ETS design, we selected the binding sequence found in the mouse Gria2 mRNA (at position 172–192 in the UTR). This choice was made based on our previous experience (Gascon et al., 2014) as well as the observations from other groups (Dutta et al., 2013; Namkung et al., 2023) having demonstrated that Gria2 is a relevant target for miR-124 in rodents and humans. For PCS, we used the complementary miR-124 sequences. For mutated versions, we replaced three nucleotides of the binding sequence from CUU to GGG. These nucleotides base pair within the seed region (position 2–4) and seve. All sequences were shown in Table 1 as well as in Supplementary Figure S1A. All sequences were cloned into the 3’ UTR of a nuclear RFP (H2B-RFP) for easy quantification using cell cytometry.

**Table 1:** Sequences used for ETS and PCS binding sequences.

Binding sequence	Nucleotide sequence
1× PCS	GGCATTCACCGCGTGCCTTA
2× PCS	GGCATTCACCGCGTGCCTTAATTCGAATGGCATTCACCGCGTGCCTTA
1× ETS	GGAACCTTCTGAGTGCCTTA
2× ETS	GGAACCTTCTGAGTGCCTTAATTCGAATGGAACCTTCTGAGTGCCTTA
1× ETS mut	GGAACCTTCTGAGTGCGGGA

### Cloning of binding sequences

Perfectly complementary sequences or endogenous sequences were cloned into a plasmid backbone containing a H2B-RFP using appropriate restriction enzymes. For miR-124 overexpression, 2 kb of the primary miR-124 were amplified from mouse genomic DNA using PCR and then cloned into a plasmid allowing the co-expression of H2B-GFP. All plasmids used here contained Tol2 sequences for the generation of stable cell lines (see below). The constructs encompassing the H2B-RFP and the different 3’UTR were amplified using PCR and cloned in an inverted orientation into a pAAV-DIO vector (Addgene #35507). Plasmids maps are shown in Supplementary Figure S4.

### Viral production

Conditional reporters AAVs were generated using pAAV-DIO plasmids described before. Synapsin-Cre AAVs were produced from Addgene plasmid #105540. Viral productions were carried out using standard protocols with slight modifications (Su et al., 2020). Briefly, HEK293T cells were transfected using the calcium phosphate method (pHelper, pRC2, pAAV; ratio 2:1:1). After 60–72 h, cells were harvested and AAV particles purified and concentrated using Takara dedicated kit (Takara, France). Viral productions were tittered using quantitative PCR (Takara, France). AAV9 serotypes of a titer around 10^12^ viral genomes/mL were used in this report. The different vectors are listed in Table 2.

**Table 2:** Viral vectors used in this study.

Name	Serotype	Titer (genomes/mL)
Syn-Cre-GFP (generated from Addgene plasmid #105540)	AAV9	2.4 × 10^12^
RFP (no binding sequences for miR-124)	AAV9	1.3 × 10^12^
miR-124 (1× ETS for miR-124)	AAV9	9.5 × 10^11^
miR-124mut (1× ETS for miR-124 mutated in 3 nucleotides pos 2–4)	AAV9	3 × 10^12^

### Construction of stable cell lines

HEK293T cells were obtained from ATCC and cultured under standard conditions (DMEM-10% Fetal Calf serum). They were used to generate cell lines stably expressing miR-124 as well as the different reporters. For that, we used a previously described procedure (Kawakami and Noda, 2004). Briefly, the different constructs were cloned into a vector containing the Tol2 sequences for the medaka fish transposase. Then, these plasmids were co-transfected with a plasmid containing the transposase into HEK cells. After a clonal dilution, cells having stably incorporated the transgene were selected by fluorescence expression. The expression of the proper construct was verified using quantitative PCR.

### Primary neuronal cultures

Cell cultures were prepared from newborn CD1 mice (postnatal day 0). Briefly, animals were decapitated, the brains rapidly transferred into ice-cold Hank’s balanced salt solution (HBSS, Gibco, France) and the different brain dissected out. Then, they are dissociated mechanically, trypsinized and purified using percoll gradient centrifugation. Cells were plated onto poly-ornithine (Sigma, France) coated 6-wells plates and allowed to grow in Neurobasal medium (Gibco) with 2% B27 supplement (Gibco), 2 mm glutamate (Gibco), 1 mm sodium pyruvate (Gibco) and antibiotics (Gibco). Cell density at plating was 3 × 10^5^ cells/well Half of the medium was replaced twice a week.

### Transfection and transduction

In all experiments with cell lines, we used Lipofectamine 3000 (Thermo) as transfection reagent following manufacturer’s recommendations. For primary cultures, AAVs particles (2 μL/well, 1 μL from the reporter AAV and 1 from the Cre AAV, representing around 10^10^ genomes/culture) were diluted in 50 μL of culture medium and then added to the cells 1 day after plating (DIV1). Neurons were analyzed 10 days after viral infection.

### Cell cytometry and FACS sorting

Cells were gently detached using trypsin and rinsed in PBS. After removal of cell clumps (70 μm cell strainer), individual cells were resuspended into 500 μL of PBS-0.1% BSA. The cells were analyzed on a cytoFlex device (Beckman Coulter) and data were analyzed with the CytoExpert software (Beckman Coulter). For FACS, infected neurons were sorted using a MoFlo Astrios EQ device (Beckman Coulter). Negative cells (either not transfected or transduced) were used to set up the gates.

### miRNA quantification

Total RNA containing miRNAs and mRNAs was then extracted from these samples using NucleoSpin miRNA kit (Machery Nagel) following manufacturer’s instructions. RNA extraction protocol includes an on-column DNase treatment (30 min). Total RNA was quantified using a NanoDrop (Fisher Scientific).

**Figure 1:**
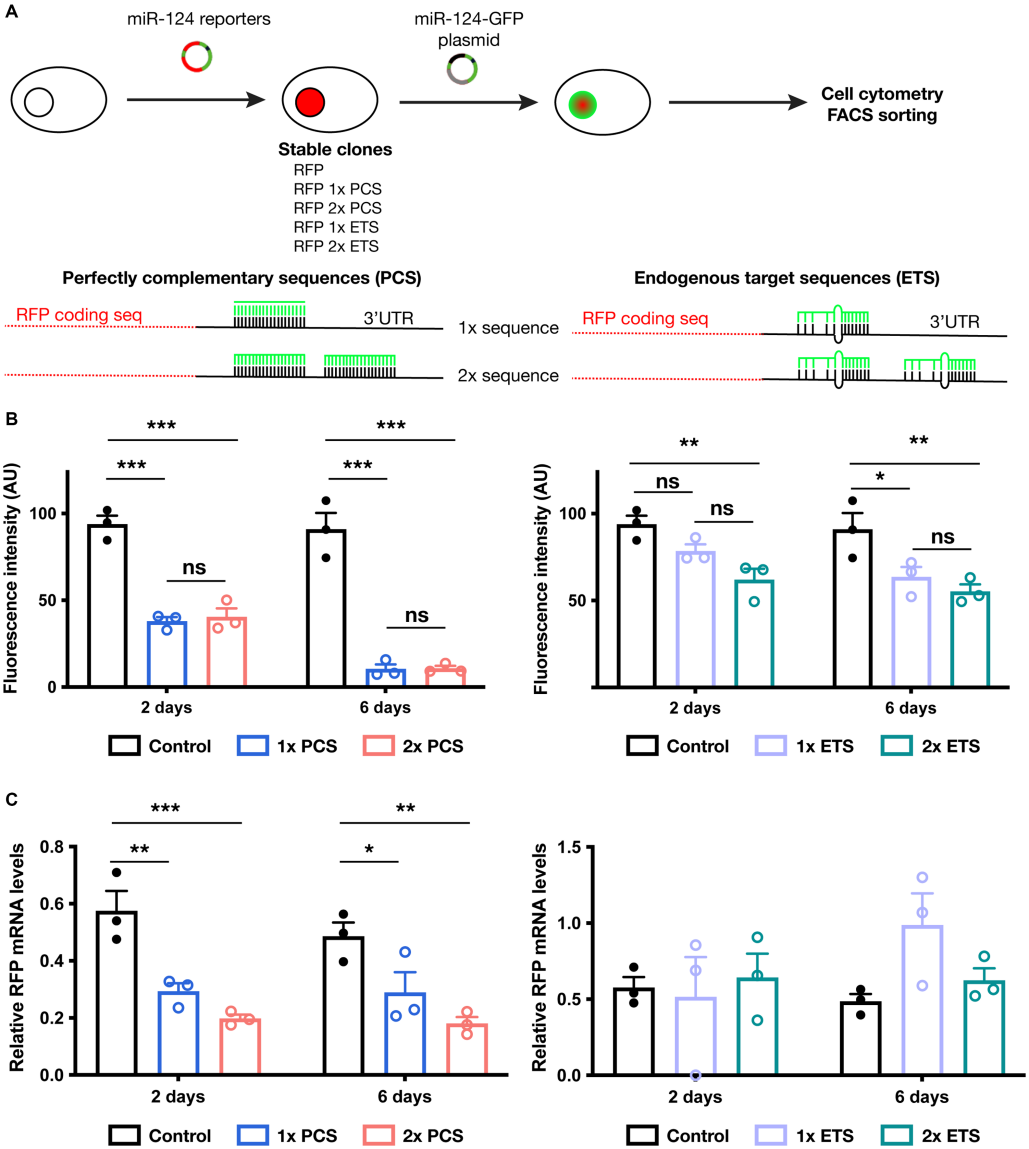
Testing reporter activity in cell lines engineered to express different miR-124 reporter constructs. **(A)** Schematic representation of experimental protocol followed in these experiments (upper panel) as well as the different miR-124 reporter designs tested (lower panels). **(B)** Effect of miR-124 transfection on RFP fluorescence in cells lines stably expressing a control reporter (RFP with no binding sequence for miR-124), PCS-containing (left panel) or ETS-containing miR-124 reporter (right panel). Cytometry analysis confirmed a reduction of RFP fluorescence in all miR-124 reporters. PCS showed a much more profound silencing effect than ETS. No significant changes were found in cells bearing 1 or 2 binding sequences. Two-way ANOVA, Tukey post-hoc test (*n* = 3 independent transfections of the different cell lines). **(C)** Effect of miR-124 transfection on RFP mRNA levels. 2 and 6 days after transient transfection with a miR-124-GFP plasmid, GFP^+^ cells were FACS sorted and the levels of RFP transcripts were measured using quantitative RT-PCR. Using GAPDH as reference gene, we observed a significant reduction of RFP mRNA only in PCS-contaning miR-124 lines. Two-way ANOVA, Holm-Sidak post-hoc test (*n* = 3 independent transfections of the different cell lines). *p < 0.05, **p < 0.01, and ***p < 0.001.

For miRNA quantification, we first performed reverse transcription using TaqMan advanced miRNA cDNA synthesis kit (Applied Biosystems, France) according to the protocol provided by the manufacturer. Abundance of miRNAs in the samples was measured using custom TaqMan probes (Applied Biosystems, United States) and a QuantStudio 7 PCR thermocycler (Applied Biosystems, France). Relative quantification was carried using the ΔΔCt method. References genes were GAPDH (Figure 1C), miR-92 (Supplementary Figure S2), miR-16 and miR-101 (Figure 3A), and miR-9 (Figure 3D). For quantification of miR-124 in FACS sorted cerebellar neurons, we did not use miR-16 or miR-101 because their expression is much lower than miR-124 and could not be consistently amplified in these samples. miR-9, a neuronal enriched miRNA, was preferred as it was the miRNA whose levels were closer to miR-124 in these cultures. All qPCR assays (including for AAV tittering) are commercially available and their references indicated in Table 3.

**Table 3:** Quantitative PCR assays used in this study.

Name	Manufacturer	Assay ID
miR-124-3p	Thermo	mmu480906_mir
miR-9-5p	Thermo	**mmu481285_mir**
miR-125a-5p	Thermo	mmu480906_mir
miR-92	Thermo	**477827_mir**
RFP	Thermo	**Mr07319438_mr**
GAPDH	Thermo	Hs99999905_m1

Name	Manufacturer	Catalog #
AAVpro Titration Kit	Takara	6,233

### Statistics

Because of the limited sample size in our experiments (*n* = 3 or 4), we could not perform normality checks. To avoid using low-power non-parametric tests (i.e., Kruskal Wallis), we transformed our data using a relative normalization method. Instead of comparing the absolute fluorescence intensity, we calculated the percentage of fluorescence in each experiment taking the RFP control sample as reference (whose intensity was aribitrarily set to 100). For the detection of significant differences among groups, we used therefore ANOVA analysis followed by post-hoc tests. Data is presented as means ± SEM unless indicated. All statistical analysis was performed using Prism GraphPad software (version 9). Details of tests and n for each experiment are provided in figure legends.

## Results

### miR-124 reporter designs based on endogenous binding sites

We generated and systematically tested *in vitro* different fluorescent reporter designs for miR-124. Our constructs encompass a nuclear RFP and different numbers (1 or 2) and types (perfectly complementary or sequences found in endogenous targets) of miR-124 target sequences (Figure 1A; Supplementary Figure S4). We first generated stable cell lines containing such reporters and evaluated how miR-124 expression influenced their activity. For that, we transiently transfected these different lines with a plasmid driving the expression of miR-124 and GFP and use untransfected cells as a reference for RFP intensity. As expected, in cells transfected with this plasmid, we did not observe any change in RFP fluorescence in GFP^−^ cells (Supplementary Figure S1A). A significant reduction of RFP fluorescence was already observed 2 days after transfection in cells expressing the perfectly complementary sequences (PCS). Silencing was exacerbated at 6 days post-transfection leading to a near complete extinction of fluorescence and confirming previous reports. The effect was similar in cells containing either one or two PCS.

In sharp contrast, reporters bearing endogenous sequences (ETS) exhibit more modest decreases in fluorescence and a slightly additive effect of increasing the number of target sequences (Figure 1B). This moderate effect might reflect a regulation comparable to that of endogenous targets.

To further understand the effect of miR-124 on the different reporters, we FACS sorted transfected cells and analyzed reporter expression at the mRNA level. Our analysis (Figure 1C; Supplementary Figure S1B) revealed striking differences of miR-124 effect on the reporters bearing ETS and PCS. Levels of RFP transcript were similar in GFP^−^ and GFP^+^ cells in the lines containing ETS reporters. Conversely, a significant reduction of RFP mRNA was found in lines bearing PCS. Together with our observations on fluorescence intensity, those results suggest that translation is inhibited for ETS reporters whereas both transcript degradation and translation blocking operate to silence PCS reporters. Interestingly, despite the fact that miR-124 levels in GFP^+^ cells increased along with time (Supplementary Figure S1C), there is no change in the abundance of RFP transcripts neither in ETS nor in PCS-bearing reporters from 2 to 6 days indicating a different time course between mRNA and protein regulation. These results indicate that ETS and PCS can be useful to estimate miR-124 expression at the protein and the mRNA level, respectively.

### miR-124 reporters bearing endogenous target sequence enable discrimination of HEK cells engineered to express different miR-124 contents

**Figure 2:**
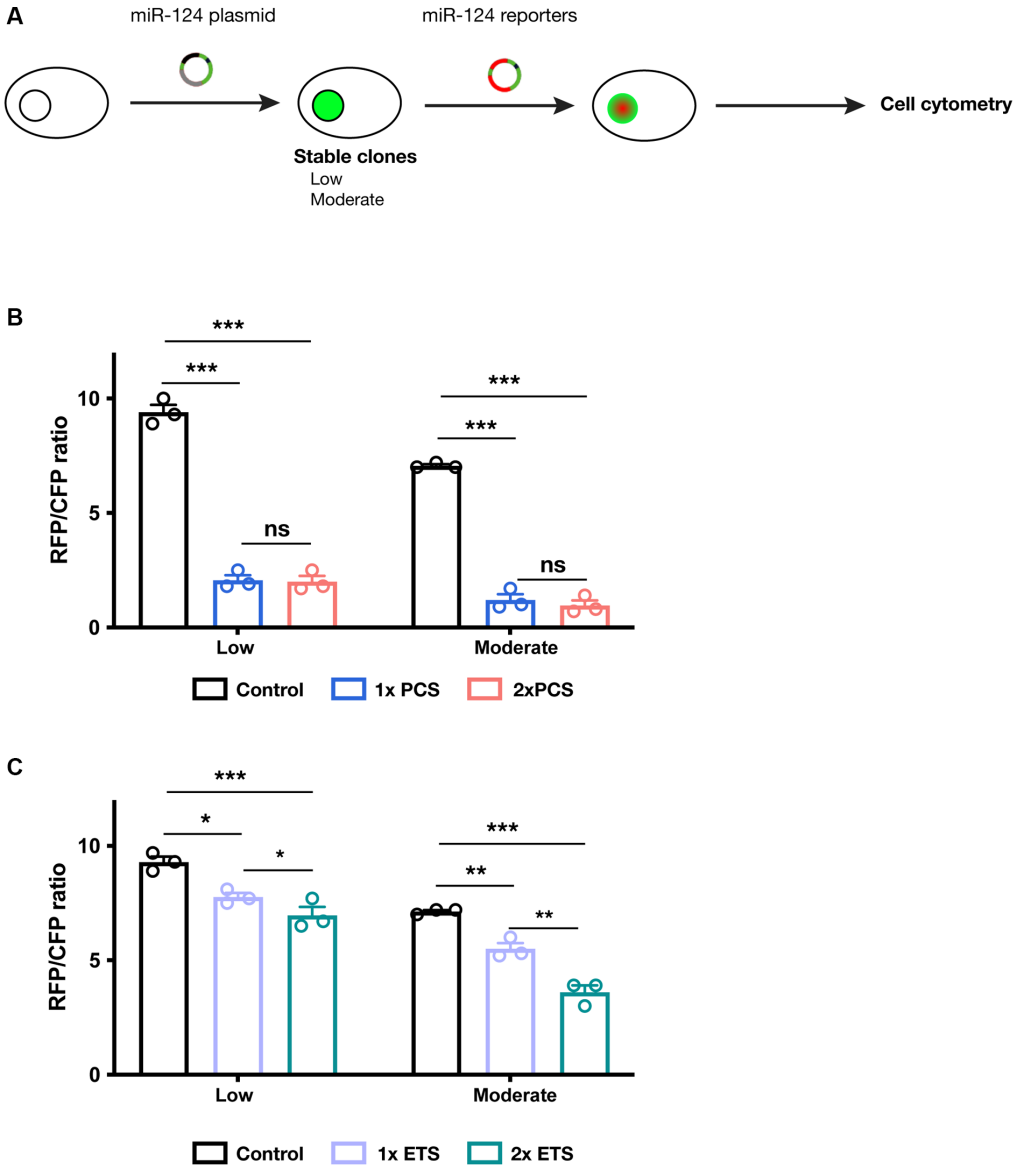
Testing miR-124 reporter designs in cell lines engineered to express different miR-124 levels. **(A)** Schematic representation of experimental protocol followed in these experiments. **(B)** Cells expressing low and moderate levels of miR-124 were transiently transfection with PCS-containing dual reporters plasmids. Cytometry analysis revealed a strong reduction of fluorescence ratio compared to cells transfected with a control plasmid. Two-way ANOVA, Tukey post-hoc test (*n* = 3 independent transfections of the different cell lines). **(C)** Similar experiments were performed using ETS-containing dual reporters. These constructs show a less intense but still significant reduction in fluorescence ratio. Two-way ANOVA, Tukey post-hoc test (*n* = 3 independent transfections of the different cell lines). *p < 0.05, **p < 0.01, and ***p < 0.001.

Since the final aim of reporters is uncovering different levels of miR-124 across different cell types *in vivo*, we explored whether our different designs can detect different miR-124 contents *in vitro* (Figure 2A). We engineered HEK cells, endogenously devoid of miR-124 (Visvanathan et al., 2007; Gascon et al., 2014; Xue et al., 2016), to stably express different amounts of this miRNA. We selected two cell lines displaying moderate but different levels of miR-124 to approach more physiological conditions (Supplementary Figure S2A).

We then analyzed the reporter activity of our different constructs in these cell lines. To avoid the intrinsic variability associated with transient transfection (in terms of copy number in each cell and plasmid dilution), we constructed dual reporter plasmids (Supplementary Figure S4) containing not only a miR-124 reporter but also a reference reporter (CFP) that serves as internal reference (Supplementary Figure S4). Using flow cytometry, we quantified the intensity of both RFP and CFP fluorescence. The RFP/CFP ratio was calculated as a reliable output of miR-124 effects on the RFP reporter. We observe that PCS containing reporters are subjected to strong inhibition (Figure 2B). As before, we found an almost identical silencing independently of the cell line and the number of binding sequences. This suggest that PCS reporter are extremely sensitive even to moderate miR-124 levels. ETS-containing reporters, in turn, exhibit a more tunable regulation and a silencing proportional to the number of sequences present in the reporter (Figure 2C). As a control, transfection of all these constructs in regular HEK cells resulted in no modification of RFP/CFP ratio (Supplementary Figure S2). Additionally, mutating 3 nucleotides of the seed region in 1× ETS reporter abrogate the regulatory effect of miR-124 arguing for the specificity of our reporters (Supplementary Figure S2). These observations together indicate that reporters containing ETS might be a more appropriate strategy to measure miR-124 activity *in vivo*.

### Optimized miR-124 reporter translates miR-124 levels in cultured neurons

**Figure 3:**
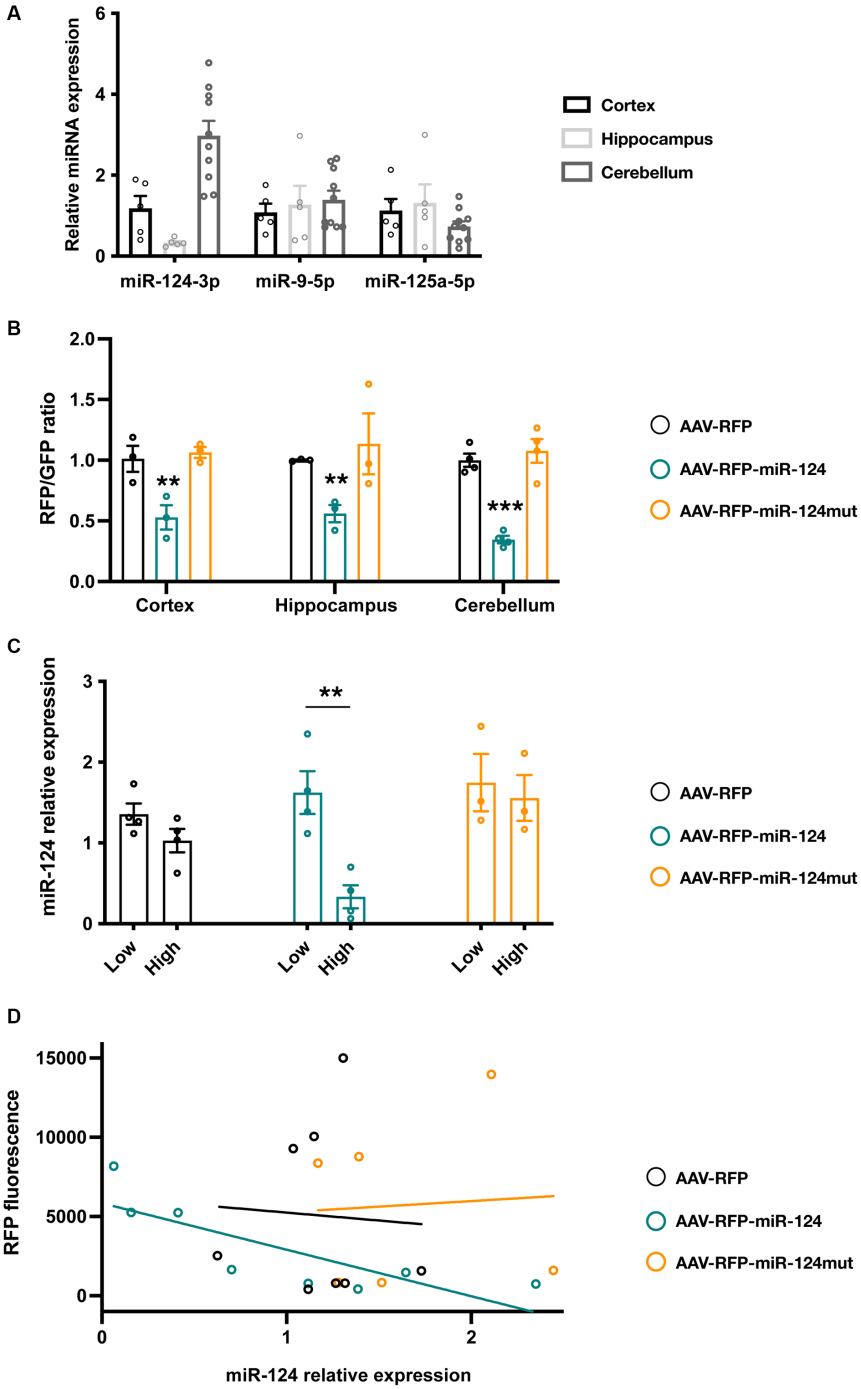
Testing reporter activity in primary neuronal cultures from cortex, cerebellum and hippocampus. **(A)** Levels of different neuronal enriched miRNAs in cortical, hippocampal and cerebellar cultures (*n* = 5 for cortical and hippocampal cultures, *n* = 10 for cerebellar cultures). **(B)** Neuronal cultures were transduced with AAVs driving the expression of different floxed-RFP reporters as well as AAV-Syn-Cre-GFP. 10 days after viral infection, intensity of the RFP (from the reporter AAV) and GFP (from Syn-Cre AAV) were measured and their ratio calculated. miR-124 reporter show a significant reduction of the ratio in all neuronal cultures but particularly in the cerebellum. Mutation of 3 nucleotides in the seed region (nt 2–4) abrogated this effect. Two-way ANOVA, Dunnett post-hoc test (*n* = 3 independent for hippocampus and cortex, *n* = 4 for cerebellum). **(C)** Analysis of miR-124 expression in cerebellar cultures transduced with AAV reporters. Using FACS, we sorted primary cerebellar neurons and quantified miR-124 expression in RFP^low^ and RFP^high^ cells taking RFP^−^ cells as reference. We showed that miR-124 levels were significantly lower in RFP^high^ neurons in cultures transduced with the AAV-RFP-miR-124. This effect cannot be observed in cultures transduced with the control AAVs (bearing a control RFP or a mutated 1× ETS reporter). Two-way ANOVA. Bonferroni post-hoc test (*n* = 4 independent for AAV-RFP and AAV-miR-124, *n* = 3 for AAV-miR-124mut). **(D)** Linear regression analysis of RFP fluorescence and miR-124 contents in cerebellar FACS-sorted neurons. When we plotted the mean fluorescence of RFP and the relative miR-124 levels, we only observed a linear relationship (*R*^2^ = 6,584) and a slope different from 0 (*p* = 0.0145) in neurons transduced with the miR-124 reporter AAV (*n* = 4 independent for AAV-RFP and AAV-miR-124, *n* = 3 for AAV-miR-124mut). *p < 0.05, **p < 0.01, and ***p < 0.001.

We next tested one of our ETS designs in a more physiological setting, primary neuronal cultures from the mouse hippocampus, cortex, and cerebellum. We selected them because, as shown in Figure 3A, cultured neurons from these brain regions express different endogenous levels of miR-124 but not of other miRNAs.

To introduce different reporters in primary neurons, we used adeno-associated vectors (AAVs) as previous work have shown their efficiency and lack of toxicity (Malik et al., 2012). We constructed three Flex conditional AAVs containing, respectively, a control reporter (no miR-124 binding sequence, AAV-RFP), a reporter bearing a single ETS (AAV-RFP-miR-124) and a mutated version (3 nucleotide mismatch in the seed region) of the reporter (AAV-RFP-miR-124mut). We transduced different neuronal cultures with the reporter AAVs along with a synapsin-Cre-GFP AAV. This strategy enables not only to drive the reporter express in similar neuronal subsets in the cultures but also to have a reference fluorescent protein from the Cre virus. We observe that AAVs transduce primary cultures with a high efficiency (ranging from 30–60% of hippocampal cells, 15–30% of cortical neurons, and 50–75% of cerebellar culture, Supplementary Figure S3A). As expected, the GFP fluorescence and the proportion of GFP+ cells does not differ among the different conditions confirming that reporter proteins are not subjected to any unspecific silencing (Supplementary Figures S3A,B). In contrast, compared to the control AAV, neurons transduced with miR-124 reporter show a marked reduction in the proportion of RFP^+^ cells (Supplementary Figure S3A) as well as in the intensity fluorescence (Supplementary Figure S3C). The intensity of such decrease varies among the brain structures analyzed. Thus, cerebellar neurons exhibit the highest silencing and hippocampal cultures are less inhibited (Figure 3B). Mutation in the miR-124 binding sequence abolishes such effect arguing for the specificity of our results. To further support these observations, we also calculated the RFP/GFP ration as a more robust quantitative measure of reporter fluorescence (Figure 3B). In the same line, we detected significant changes only in neurons transduced with the miR-124 reporter AAV.

To confirm these observations and to avoid the variability among different experiments, we sought to analyze the activity of our reporters in neurons from the same culture. We reasoned that, if our reporter fluorescence is negatively correlated to miR-124 contents, neurons displaying high RFP levels (RFP^high^) might contain lower amounts of miR-124 relative to those in which RFP silencing is stronger (RFP^low^). Conversely, in neurons transduced with the control or mutated reporter, miR-124 levels should be no correlated to fluorescence intensity. We therefore transduced cerebellar neurons (those showing highest miR-124 expression and the highest transduction efficiency), carried out FACS sorting to isolate RFP^high^ and RFP^low^ neurons and measured miR-124 in these different populations. We first confirmed that, in neurons transduced with the miR-124 reporter, there was an obvious left-shift in the fluorescence distribution that resulted in a net decrease of RFP^high^ cells (Supplementary Figure S3C). Regarding miR-124 contents, neurons having received the control or mutated construct show similar miR-124 contents irrespective of the fluorescence intensity (Figure 3C). In contrast, in those transduced with miR-124 reporter, RFP^low^ neurons exhibit a significant enrichment in miR-124 levels compared to RFP^high^ neurons suggesting that our reporter is subjected to miRNA silencing and its levels inversely correlated to miR-124 (Figure 3B). Finally, using linear regression, we sought to confirm the inverse correlation of reporter fluorescence and miR-124 contents in FACS-sorted cerebellar neurons. For that, we plotted the mean RFP fluorescence and relative miR-124 levels in each condition. As shown in Figure 3D, linear correlation was remarkably good for the miR-124 reporter (*R*^2^ = 0.6584) but rather poor for the control RFP reporter as well as in the mutated reporter (*R*^2^ = 0.01877 and *R*^2^ = 0.004265, respectively). More importantly, the slope analysis revealed a significant difference from 0 only for miR-124 reporter (*p* = 0.01255) but not the control reporters (*p* = 0.8955 and 0.9022). These findings provide further support for the inverse relationship between miR-124 abundance and reporter fluorescence. Overall, our *in vitro* experiments support the notion that optimized miR-124 reporters could be useful for *in vivo* applications.

## Discussion

In this work, we optimized an existing technique (fluorescent reporters) to infer miR-124 levels *in vitro*. Our approach relies on the expression of a nuclear reporter fluorescent protein bearing endogenous binding sequences for miR-124. Our reporters showed a moderate, more physiological silencing which enables the detection of miR-124 differences among distinct neuronal types in culture. This is in sharp contrast to recent studies in which miR-124 reporters (or reporters for other miRNAs) are submitted to strong inhibition (Åkerblom et al., 2012, 2013; Akerblom et al., 2014). Our results show an important difference in the mechanism of silencing among sequences; PCS destabilizes the transcript and block protein translation whereas ETS do only act at the translational level. These results are in line with previous work indicating that endonucleolytic cleavage is favored by perfect base-pairing between the miRNA and the mRNA (Yekta et al., 2004; Valencia-Sanchez et al., 2006) and might explain the striking different levels of inhibition across reporters bearing PCS and ETS sequences.

Although intensively studied, our understanding of miR-124 expression levels across cell types remains rudimentary. The main aim of this study is to generate miR-124 reporters that could help unraveling the precise expression pattern of this miRNA in the brain. More precisely, miR-124 reporters are intended to: (i) estimate the abundance of this miRNA in living cells and (ii) compare expression among different neuronal subsets. Concerning the first point, this is a key aspect as most techniques aiming at measuring and comparing miRNAs expression levels are end-point techniques (quantitative PCR, miRNA sequencing, *in situ* hybridization or miRNA microarrays). This is precluding the prospective identification of specific neuronal subtypes according to the expression of a particular miRNA and the further investigation of how changes in this miRNA alter neuronal properties in different cellular contexts. Regarding the expression of a particular miRNA in precise cell types, we have provided evidences that miRNA reporters could be packed in Cre-dependent AAVs and delivered to specific cell subsets so that, in combination with transgenic Cre lines, they can be used to thoroughly map the expression of miR-124 during development, aging or in pathological conditions.

In this work, we provided initial evidence that reporters bearing ETS could be more appropriate to estimate miR-124 levels *in vitro*. The *in vivo* application of approaches described here is more challenging. These experiments rely on the stereotactic injection of the reporter AAV into a target brain area. This approach introduces important bias in the number of transduction units delivered to each cell as the number of viral particles exponentially decrease with the distance. Thus, those cells near the injection site would receive high number of particles potentially driving reporter levels out of the regulatory range of miR-124 *in vivo*. Conversely, those far from the injection might receive suboptimal number of AAVs particles so that reporter expression after miR-124 silencing does not reach detection thresholds. To circumvent such limitations, *in vivo* applications would require an optimization of viral constructs: (i) promoter choice so that the reporter levels are appropriate independently of the number of AAV copies in each cell; (ii) including a second reporter not subjected to miR-124 silencing so that it can be used as internal reference for AAV infection. *In vivo* validation of miR-124 reporters will be a focus of our future work.

Research on miRNAs has been exponential in the last two decades. This interest has been boosted, in part, by the paradigm shift in pharmacological approaches against complex human disorders such as neurodegenerative diseases (Millan, 2017; Wang et al., 2018; Lee et al., 2021; Nguyen et al., 2022). Rather than looking for one target, current therapeutic strategies aim at targeting multiple pathways shared by a number of conditions (Ibrahim and Gabr, 2019; Grosjean et al., 2022). miRNA-based therapy is particularly suited for such purposes, as their mechanism of action is grounded on regulation of multiple components across molecular cascades that might converge on disease-relevant alterations (Kosik and Krichevsky, 2005). Although promising, application of miRNAs to the clinic requires further fundamental research to better characterize not only how miRNAs target key pathological pathways but, more importantly, which are the cells types where they should be delivered. New tools are required not only to increase our understanding of miRNA expression patterns in the brain but also to monitor cell-specific changes in miR-124 levels in animal models of neurodegenerative disorders (Tran et al., 2023; Zhao et al., 2023). Our work opens a potential new option for the design of such tools.

A major limitation of our study concerns the reporter design. Since miRNA binding silence the expression of the target mRNA, our reporter is inversely correlated to the actual miRNA levels. A reporter showing a direct relationship to the miRNA contents might be preferable in certain contexts. In addition, it remains to be determined whether reporter bearing one target endogenous sequence are functional for other miRNAs. Despite these limitations, reporters tested here should provide a rationale for future designs and hold a great potential to unravel miR-124 expression in the brain.

## Data availability statement

The original contributions presented in the study are included in the article/Supplementary material, further inquiries can be directed to the corresponding author.

## Author contributions

CL: Data curation, Investigation, Writing – original draft, Formal analysis. CR: Formal analysis, Investigation, Writing – original draft, Conceptualization. FJ: Conceptualization, Investigation, Writing – original draft, Methodology. AB: Investigation, Writing – review & editing. EC-G: Investigation, Writing – review & editing. NP: Investigation, Writing – review & editing. EG: Investigation, Writing – review & editing, Data curation, Funding acquisition, Methodology, Project administration, Supervision, Writing – original draft.
