# Supplementary material for: Optimized miR-124 reporters uncover differences in miR-124 expression among neuronal populations in vitro
Source: Front Neurosci. 2023 Oct 18;17:1257599. doi: 10.3389/fnins.2023.1257599 (PMC10619730; doi:10.3389/fnins.2023.1257599)

Supplementary Figure legends

Supplementary Figure 1. Testing reporter activity in cell lines engineered to express different miR-124 reporter constructs.

A. Left: schematic illustration of the different reporter 3' UTR and the binding of miR-124 to it. The seed region of miR-124 are highlighted in gray
   Right: conservation of miR-124 binding sequence in Gria2 transcripts across mammals.
B. RFP fluorescence in cells lines stably expressing a control reporter is not modified in GFP^- cells (control for experiments in Figure 1C). n=3 independent experiments.
C. Illustration of FACS sorting gating strategy for experiments in Fig 1c. Untransfected cells (left) were used to set up the sorting-windows.
D. In transient transfection experiments, miR-124 levels in GFP^+ cells increased over time. No significant difference can be observed across reporter cell lines. n=3 independent experiments.

A **Lepolard et al Suppl Figure 1**

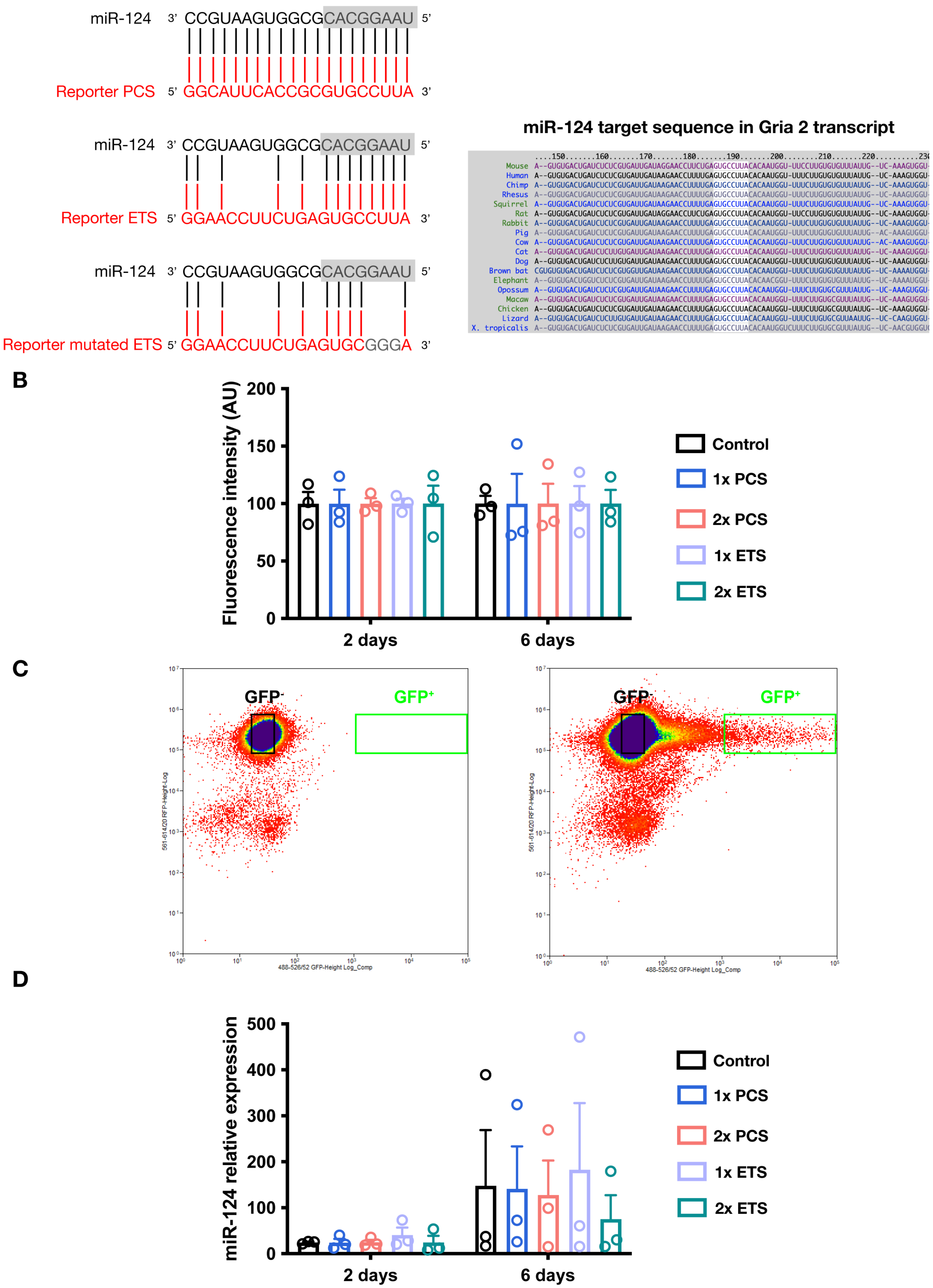

Supplementary Figure 2. Control experiments for transient transfection with dual reporters.

A. Quantification of miR-124 in HEK293T cells stably expressing this miRNA. As a reference, we used the levels of miR-124 in transient transfection experiments. Our stable cell lines display much lower levels of miR-124 (about 25% and 10% of the reference). n=2 independent experiments.
B. In HEK293T cells (devoid of miR-124) no downregulation of any miR-124 reporter can be observed. n=2 independent experiments.
C. Mutating 3 nucleotides in the seed region of the 1x ETS reporter abrogate the effect observed in HEK293T cells expressing low or moderate levels of miR-124. n=3 independent experiments.

A

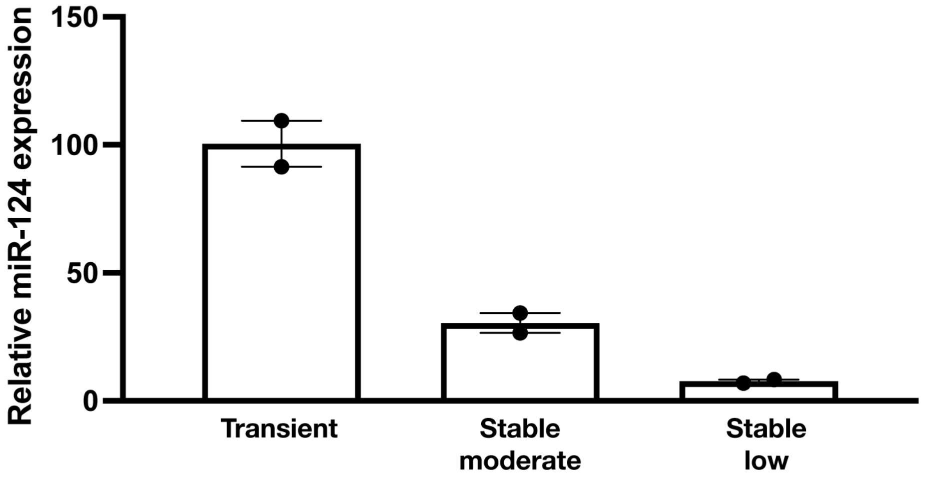
Relative miR-124 expression
150
100
50
0
Transient
Stable
moderate
Stable
low

B

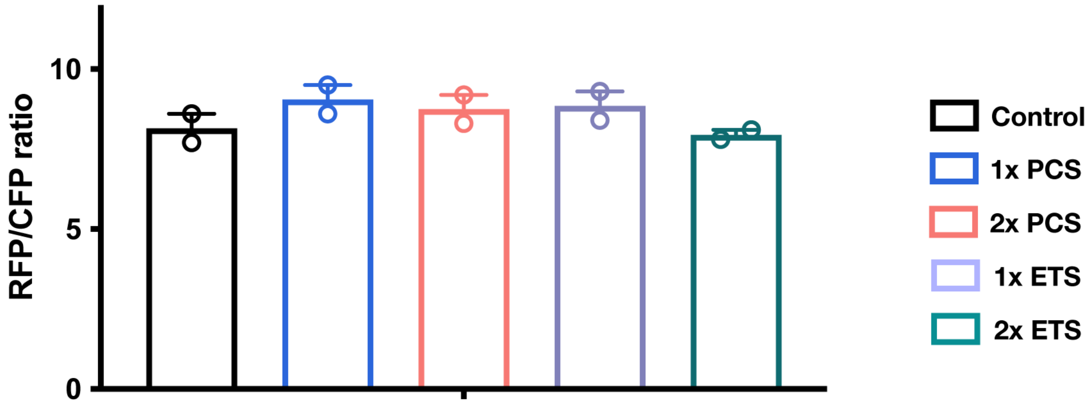
RFP/CFP ratio
10
5
0
Control
1x PCS
2x PCS
1x ETS
2x ETS

C

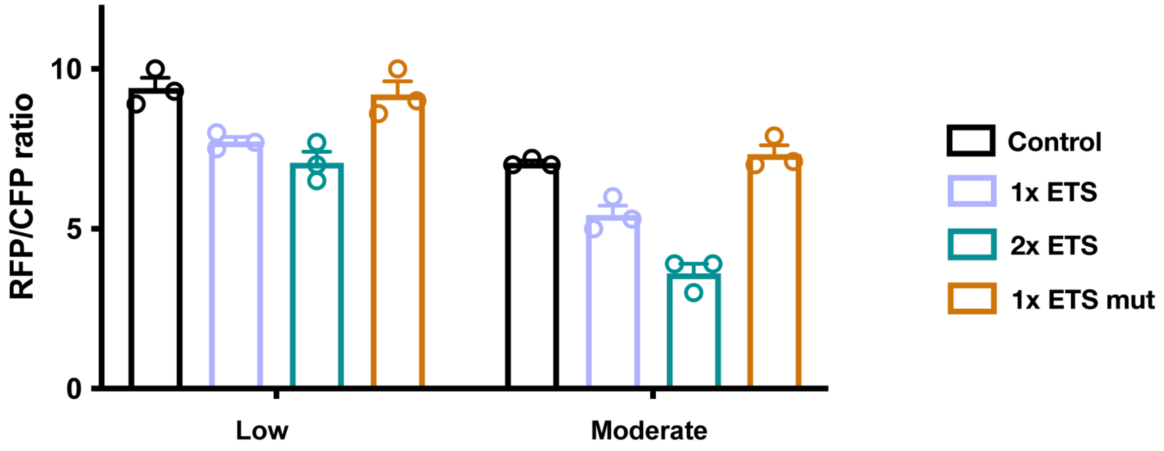
RFP/CFP ratio
10
5
0
Low
Moderate
Control
1x ETS
2x ETS
1x ETS mut

Supplementary Figure 3. Testing reporter activity in primary neuronal cultures from cortex, cerebellum and hippocampus.

A. Proportion of RFP^+ and GFP^+ cells in primary neurons coming from different brain structures. (n=3 independent for hippocampus and cortex, n=4 for cerebellum).

B. RFP and GFP levels in neuronal cultures transduced with AAV-RFP reporters. Whilst GFP fluorescence showed no change across conditions, RFP was strongly downregulated in neuronal cultures transduced with the miR-124 reporter. Two-way ANOVA, Dunnett post-hoc test (n=3 independent for hippocampus and cortex, n=4 for cerebellum).

C. Gating strategy in primary cerebellar neurons infected with different AAV-RFP reporters for quantification of miR-124. Of note, in cultures infected with the AAV-RFP-miR-124, a leftward switch is observed in the fluorescence intensity.

Lepolard et al Suppl Figure 3

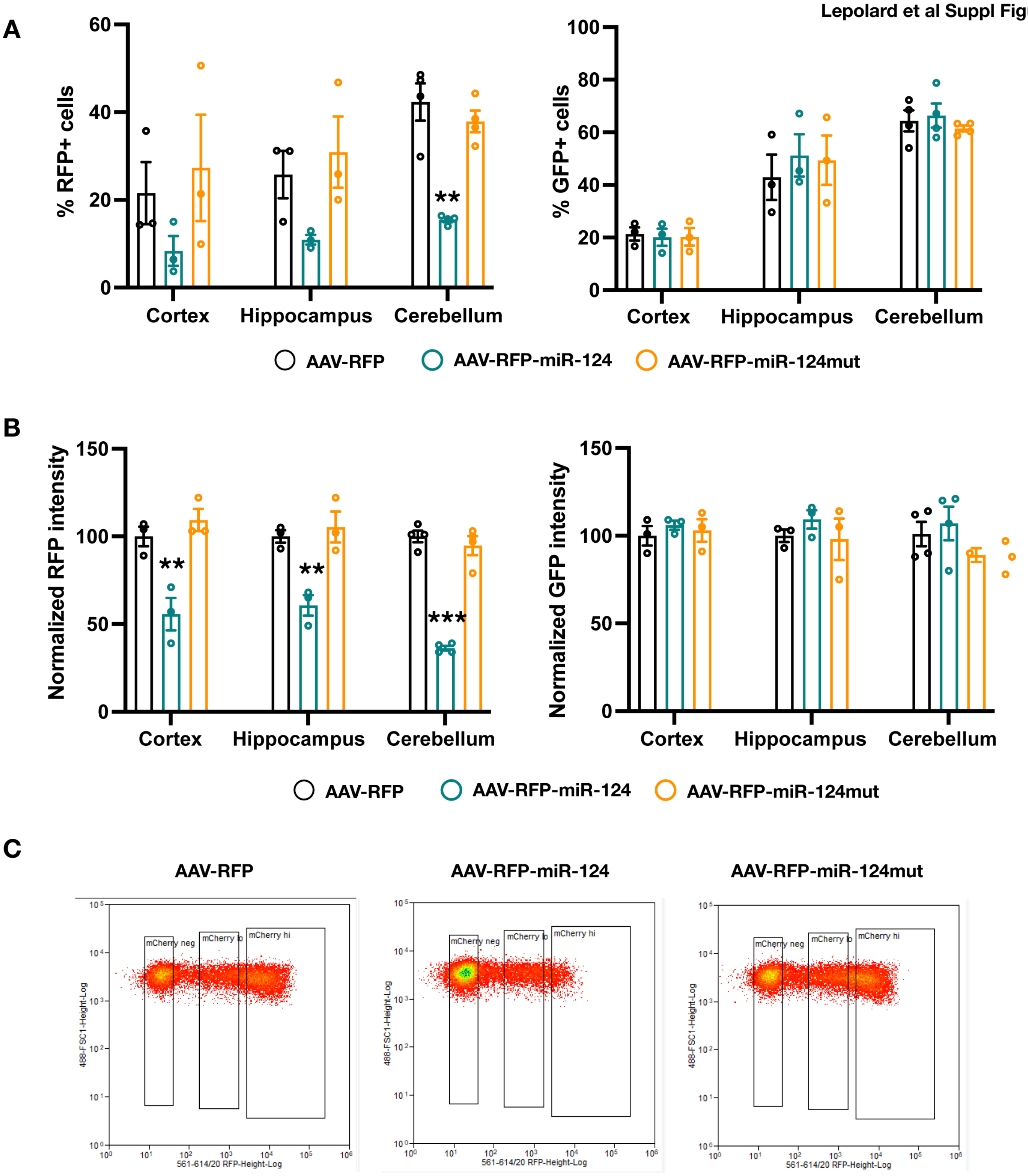

Supplementary Figure 4. Schematic illustration of plasmid maps used in this study.

Lepolard et al Suppl Figure 4

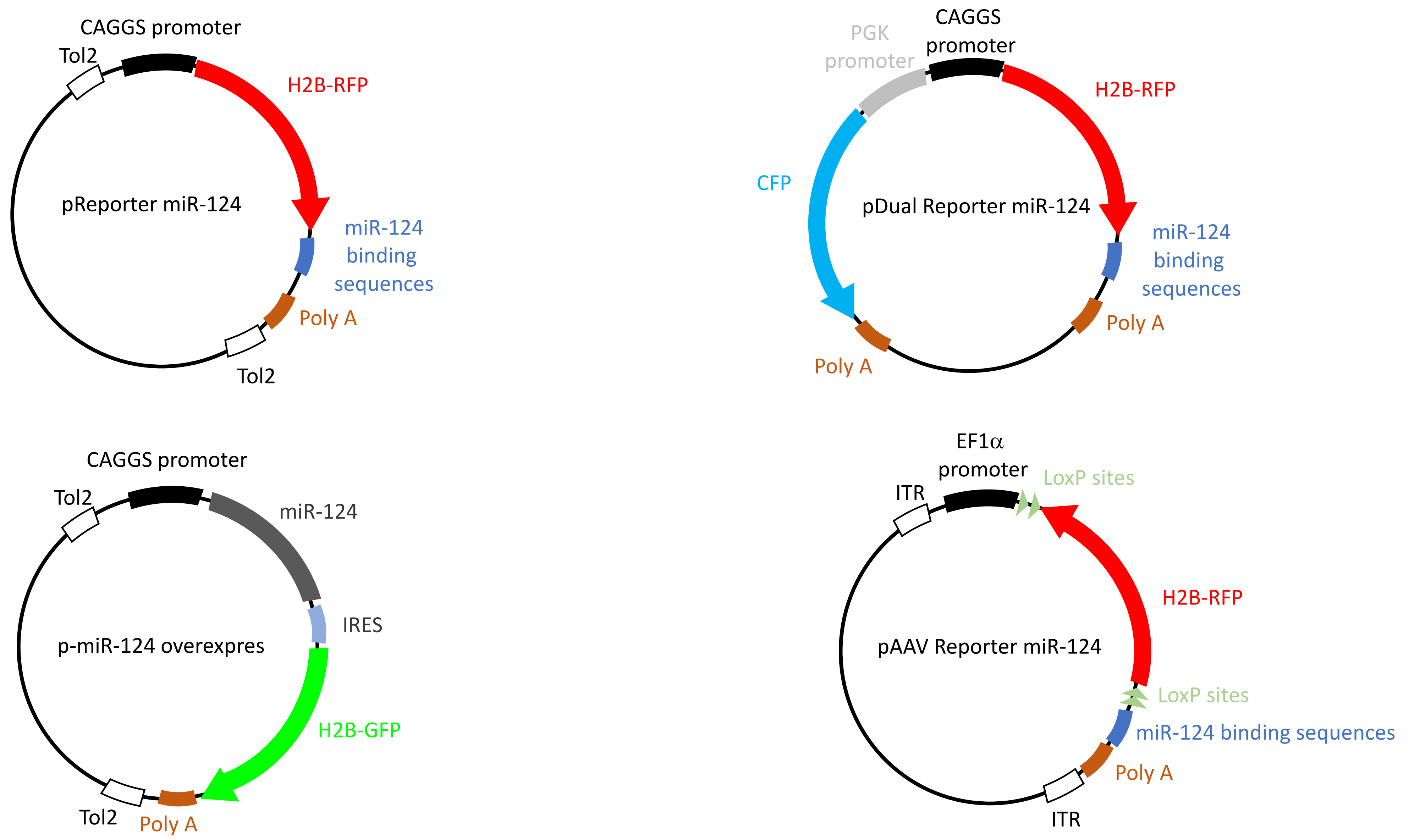

Supplement: Supplementary file 1 [file Data_Sheet_1.pdf]
